# *Notes from the Field:* Rabies Exposures from Fox Bites and Challenges to Completing Postexposure Prophylaxis After Hurricane Irma — Palm Beach County, Florida, August–September 2017

**DOI:** 10.15585/mmwr.mm6836a4

**Published:** 2019-09-13

**Authors:** Briana O’Sullivan, Ryan Burke, Denise Bassaline

**Affiliations:** ^1^Florida Department of Health in Palm Beach County; ^2^Zoonosis Control Branch, Texas Department of State Health Services.

On August 29, 2017, epidemiology staff members at the Florida Department of Health in Palm Beach County (DOH-Palm Beach) were notified through syndromic surveillance via the Florida Electronic Surveillance System for the Early Notification of Community-based Epidemics (ESSENCE-FL) of an emergency department visit at hospital A for a fox bite received by a county resident (patient A) on August 27. ESSENCE-FL is a system that includes syndromic surveillance that allows users to query emergency department (ED) visit records electronically to conduct surveillance for hospital visits related to reportable conditions. ESSENCE-FL provides data that are deidentified but include patient demographic information along with a patient identification number allowing system users to identify cases of reportable conditions that might not otherwise have been reported through ED visit records. According to medical records, a bite to the foot occurred while the patient, who was experiencing homelessness, was sleeping outdoors. At that time (day 0), patient A received rabies postexposure prophylaxis (rPEP), including wound washing, human rabies immune globulin, and dose 1 of 4 doses of rabies vaccine (Figure), with subsequent doses to be administered on days 3, 7, and 14 (*1*). On August 29, Palm Beach County Animal Care and Control (PB-ACC) informed DOH-Palm Beach of a second person (patient B) bitten by a fox on August 28. While interviewing patient B outside of his workplace, PB-ACC euthanized an aggressive gray fox suspected of causing the bites and sent it to the DOH Bureau of Public Health Laboratories in Jacksonville for testing. On August 30, laboratorians reported that brain tissue from the fox tested positive for rabies by direct fluorescent antibody testing (*2*).

**FIGURE:**
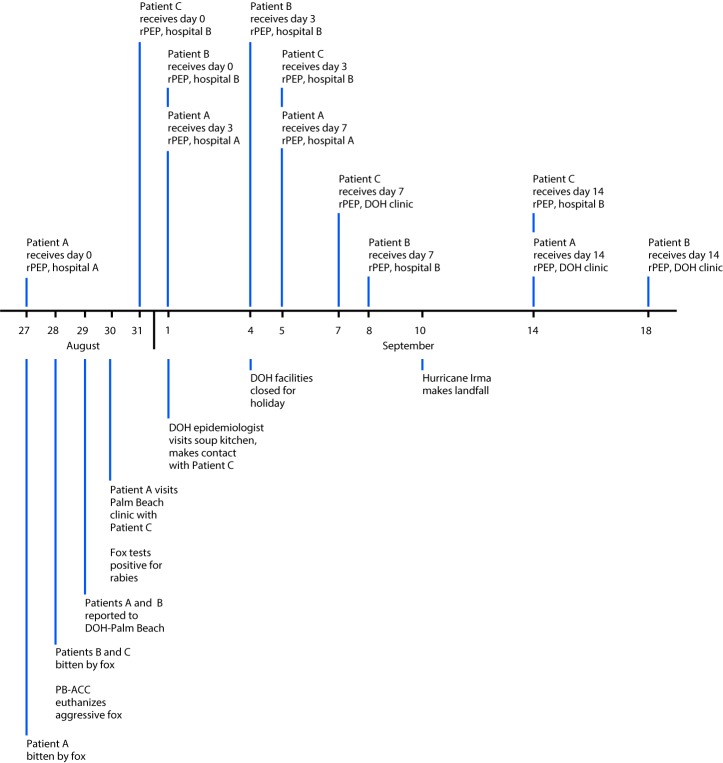
Timeline of events surrounding fox bites and receipt of rabies postexposure prophylaxis* for three patients — Palm Beach County, Florida, August–September 2017 **Abbreviations:** DOH = Florida Department of Health; PB-ACC = Palm Beach Animal Care and Control; rPEP = rabies postexposure prophylaxis. * rPEP consists of wound washing, 1 dose of human rabies immune globulin (on day 0), and 4 doses of rabies vaccine (on days 0, 3, 7, and 14).

On August 30, patient A visited a DOH-Palm Beach clinic for the second rabies vaccine dose, accompanied by a third person bitten by a fox (patient C) who was previously unknown to DOH-Palm Beach and PB-ACC. Neither of these two patients had a referral to the clinic, and both left before receiving vaccine. No contact information was collected, although both patients were reported by clinic staff members to be experiencing homelessness. Although patient B was initially interviewed by PB-ACC, DOH-Palm Beach had difficulty contacting the patient to explain the need for rPEP. After multiple attempts, patient B was contacted by DOH-Palm Beach through the patient’s employer on September 1 and subsequently initiated rPEP at hospital B.

On September 1, DOH-Palm Beach visited a soup kitchen in an urban area near where the rabid fox had been found to search for patients A and C. Patient C was contacted there and reported that rPEP had been initiated at hospital B on August 31. Contact information was exchanged, and the patient received a vaccination schedule. Patient A received vaccine dose 2 on September 1 after contacting DOH-Palm Beach using information obtained from Patient C.

Because of office closures and transportation difficulties caused by Hurricane Irma, all three patients experienced modifications to their rabies vaccination schedules. Once initiated, rPEP should be kept as close to schedule as possible, although delays in vaccine administration of up to a few days are not considered likely to have a significant adverse effect (*3*). DOH facilities were closed on September 4 for a state holiday, and patients with doses due that day were advised to go to the hospital to remain on schedule. Patient B received rabies vaccine doses 2 and 3 at hospital B on September 4 and September 8, respectively. Patient C received vaccine dose 2 at hospital B (September 5), and dose 3 at a DOH clinic (September 7). Patient A received vaccine dose 3 at hospital A (September 5).

On September 10, Hurricane Irma made landfall in southern Florida. DOH-Palm Beach suspended services at clinics and offices on September 8 and reopened with limited services on September 13. On September 14, patients A and C received rabies vaccine dose 4 at a DOH clinic and hospital B, respectively. Patient B received vaccine dose 4 at a DOH clinic on September 18.

Possible rabies exposure is a reportable condition in Florida; however, these cases were not reported to DOH-Palm Beach by health care providers even though fox bites are considered high-risk exposures (*4*). Surveillance through ESSENCE-FL not only provided the initial notification for this investigation to DOH-Palm Beach, but a method to track patients’ hospital visits for rPEP when they received care outside of health department clinics. This was important in the days following Hurricane Irma, when DOH-Palm Beach offices were closed and patients had rPEP scheduled. Epidemiologists were able to log into ESSENCE-FL remotely to monitor patient visits using medical record numbers or patient demographics. ESSENCE-FL monitoring helped DOH-Palm Beach identify missed rPEP visits and facilitated contact with patients to ensure receipt of recommended doses. All three patients completed their rPEP series by September 18, 2017, with schedule modifications. Subsequently, no human rabies cases associated with these exposures were reported in Palm Beach County.

## References

[1] Rupprecht CE, Briggs D, Brown CM, Use of a reduced (4-dose) vaccine schedule for postexposure prophylaxis to prevent human rabies: recommendations of the Advisory Committee on Immunization Practices. MMWR Recomm Rep 2010;59(No. RR-2). 20300058

[2] Ma X, Monroe BP, Cleaton JM, Rabies surveillance in the United States during 2017. J Am Vet Med Assoc 2018;253:1555–68. 10.2460/javma.253.12.1555 30668262

[3] Manning SE, Rupprecht CE, Fishbein D, . Human rabies prevention—United States, 2008: recommendations of the Advisory Committee on Immunization Practices. MMWR Recomm Rep 2008;57(No. RR-3). 18496505

[4] Florida Department of Health. Rabies prevention and control in Florida: rabies exposure and risk assessment. Tallahassee, FL: Florida Department of Health; 2016. http://www.floridahealth.gov/diseases-and-conditions/rabies/_documents/rabies-prevention-and-control-guidebook/rabies-exposure-and-risk-assessment.pdf

